# Role of Systematic Biopsy in the Era of Targeted Biopsy: A Review

**DOI:** 10.3390/curroncol31090383

**Published:** 2024-09-03

**Authors:** Wojciech Malewski, Tomasz Milecki, Omar Tayara, Sławomir Poletajew, Piotr Kryst, Andrzej Tokarczyk, Łukasz Nyk

**Affiliations:** 1 Second Department of Urology, Centre of Postgraduate Medical Education, 02-511 Warsaw, Poland; o.tayara@wp.pl (O.T.); slawomir.poletajew@cmkp.edu.pl (S.P.); piotr.kryst@onet.pl (P.K.); andtok00@gmail.com (A.T.); ukinyk@poczta.fm (Ł.N.); 2 Department of Urology, Poznan University of Medical Sciences, 61-701 Poznan, Poland; mileckito@gmail.com

**Keywords:** prostate cancer, systematic biopsy, targeted biopsy, MRI-targeted biopsy, magnetic resonance imaging (MRI), biopsy techniques, prostate cancer management

## Abstract

Prostate cancer (PCa) is a major public health issue, as the second most common cancer and the fifth leading cause of cancer-related deaths among men. Many PCa cases are indolent and pose minimal risk, making active surveillance a suitable management approach. However, clinically significant prostate carcinoma (csPCa) can lead to serious health issues, including progression, metastasis, and death. Differentiating between insignificant prostate cancer (inPCa) and csPCa is crucial for determining appropriate treatment. Diagnosis of PCa primarily involves trans-perineal and transrectal systematic biopsies. Systematic transrectal prostate biopsy, which typically collects 10–12 tissue samples, is a standard method, but it can miss csPCa and is associated with some complications. Recent advancements, such as magnetic resonance imaging (MRI)-targeted biopsies, have been suggested to improve risk stratification and reduce overtreatment of inPCa and undertreatment of csPCa, thereby enhancing patient quality of life and treatment outcomes. Guided biopsies are increasingly recommended for their ability to better detect high-risk cancers while reducing identification of low-risk cases. MRI-targeted biopsies, especially when used as an initial biopsy in biopsy-naïve patients and those under active surveillance, have become more common. Utilization of MRI-TB alone can decrease septic complications; however, the combining of targeted biopsies with perilesional sampling is recommended for optimal detection of csPCa. Future advancements in imaging and biopsy techniques, including AI-augmented lesion detection and robotic-assisted sampling, promise to further improve the accuracy and effectiveness of PCa detection.

## 1. Introduction

Prostate cancer (PCa) is a significant public health concern, since it was the second most prevalent cancer and the fifth leading cause of cancer-related mortality among men in 2020, contributing to over 375,000 deaths globally during this period [1]. Estimated cancer-related mortality amounts to 11%; however, the 5-year relative survival rate for prostate cancer has increased from the mid-1970s to reach 97% in 2024 [2]. PCa is heterogeneous in terms of morphological, genetic, and clinical features and contains various morphologically and clonally distinct foci. A considerable portion of PCa cases exhibit indolent behaviour, posing minimal threat if left unidentified [3]. In case of indolent cancers, active surveillance appears to be a sufficient management mode. Conversely, clinically significant prostate carcinoma (csPCa) has substantial therapeutic implications, including progression, metastasis, and potential PCa-specific mortality [4]. The term csPCa describes a form of prostate cancer with the potential to result in mortality of the affected patient and it is used to distinguish it from tumor variants typically lacking such lethality [5]. However, the definition of csPCa includes features extending beyond histological grading to encompass individual patient factors, therefore precise specification of csPCa poses a challenge and differs in the available literature. Typically, the criteria rely on histological parameters, such as the Gleason grading system or the International Society of Urological Pathology (ISUP) grade systems [6,7]. Additional parameters, including PSA levels, race, family history, or cancer volume, may also be included in the classification [8,9]. Prostate-specific antigen (PSA) screening has contributed to the decrease in PCa mortality, but also poses the risk of overdiagnosis (since it can be increased also in benign prostatic hyperplasia, prostatitis and other non-malignant conditions) and consequent complications from unnecessary treatment [10,11]. Discrimination between clinically insignificant prostate cancer (inPCa) and csPCa is crucial in determining prostate cancer lethality [12]. However, risk stratification during diagnosis is complicated by inter- and intra-tumoral heterogeneity.

Systematic transrectal prostate biopsy serves as a standard method for the collection of tissue samples to confirm the diagnosis in patients with clinical suspicion of PCa based on elevated levels of PSA and/or abnormal findings on digital rectal examination (DRE) [13,14]. This type of prostate biopsy typically involves the systematic collection of 10–12 biopsy cores from both lobes of the prostate [5,15]. However, since this diagnostic tool has been suggested to miss csPCa and to be associated with complications, new techniques have been developed. Magnetic resonance imaging (MRI) has emerged as a valuable tool in diagnostics since it facilitates risk stratification of visible lesions based on diffusion and perfusion patterns and enables targeted biopsy needle placement [16]. MRI-targeted biopsies have been suggested to enhance the detection rates of clinically relevant PCa; however, it is the approach combining targeted and systematic biopsies that appears to offer optimal sensitivity for cancer detection [17].

This review aims to assess the evolving role of systematic biopsy in the era of targeted biopsy approaches in prostate cancer diagnosis.

## 2. Systematic Biopsy

Systematic transrectal ultrasound (TRUS)-guided biopsy, which has been widely used globally for decades, has progressed from sextant templates to 12-core biopsy schemes [18]. This diagnostic procedure involves sampling the peripheral zone of the prostate, where cancer is predominantly located, with 8 to 12 cores (maximum 19 cores, but most commonly 10–12) depending on prostate size [15,19]. The utilization of more comprehensive biopsy schemes, including the lateral and peripheral aspects of the prostate with increased number of sampling sites, translates into higher detection rates and reduced variation in PSA-related detection of cancer [18]. TRUS biopsy primarily relies on ultrasound use for anatomical guidance (identification of the prostate) since suspicious lesions are generally not visible using this imaging technique, potentially resulting in random and systematic errors, as well as missing significant cancers [19]. Due to variations in tumor differentiation within various areas of the prostate, sampling errors may result in under-grading [20]. The estimated false-negative rate of systematic biopsy performed in the case of any cancer is between 25% and 40% [21]. Misclassification is frequently associated with inadequate sampling of cancer lesions at their greatest diameter or highest grade [22,23].

Two primary approaches, = biopsy and transrectal biopsy with varying core numbers, are primarily used. Transrectal biopsy is typically conducted as a quick, in-office procedure under local anesthesia [24]. It involves multiple passes of the biopsy needle through the rectal mucosa, which may increase the risk of infections [25]. Extended biopsy protocols include cores from the lateral peripheral zone and those from lesions detected through palpation or imaging [26]. In turn, transrectal saturation biopsy, involving comprehensive sampling (over 20 prostate biopsies) frequently overlooks anterior zones, which diminishes its accuracy [27]. Moreover, such intensified approaches are less common due to their burden on patients, elevated complication rates, and tendency to over-diagnose insignificant prostate cancer [28]. Saturation biopsies are mostly reserved for instances of repeat biopsy procedures [26]. It appears that increasing core numbers through saturation techniques may reveal cancers overlooked during extended core sampling. However, this approach also elevates the risk of over-detecting indolent cancers without substantially enhancing cancer detection rates (CDR) or pathology concordance [29].

The trans-perineal approach for prostate biopsy serves as an alternative to the conventional transrectal route. Its advantages include improved access to the anterior aspect of the prostate gland, reduced incidence of infectious complications, and the potential for refining the three-dimensional localization and documentation of sampling through the utilization of a biopsy template with coordinates [30]. Trans-perineal biopsy may be either executed in the operating room under general or as an office procedure with local anesthesia, though the first approach is now rarely used [24]. During such biopsy, the needle is guided longitudinally along the axis of the prostate, improving anterior prostate sampling [31]. This technique has demonstrated greater capacity for detecting clinically significant prostate cancer and for upgrading men under active surveillance [32]. Moreover, trans-perineal biopsy avoids the transfer of rectal flora into the prostate and bloodstream, thus posing lower risks of infectious complications [33]. Therefore, current European Association of Urology guidelines strongly recommend performing prostate biopsy using the trans-perineal approach due to the lower risk of infectious complications [34]. However, the recent PREVENT randomized trial demonstrated that transrectal biopsy with targeted prophylaxis shows comparable infection rates to office-based trans-perineal biopsy [35]. The American Urological Association guidelines suggest that clinicians may use either a transrectal or trans-perineal biopsy route when performing a biopsy (conditional recommendation; evidence level: grade C) [36].

Template grid is frequently used for biopsy guidance, which increases procedural time and medical costs [37]. However, due to the lack of consensus on a template for - prostate biopsy in terms of the optimal number and location of biopsy cores, various types of grids have been developed, including the Barzell template, which comprises 26–35 cores. The introduction of the freehand technique has decreased anesthesia requirements from general to local or local/sedation [38]. In this technique a common access cannula is introduced through the perineal skin, allowing repeated passage of the biopsy needle through two needle puncture sites, one on each side of the perineum [24,39] This approach facilitates biopsy performance in office settings and reduces needle punctures through the skin. Some studies suggested a correlation between core number and csPCa detection, while other failed to observe such relationship [40,41,42]. In general, the number of biopsy cores should depend on prostate volume to avoid false-negative results. Previous guidelines of the European Association of Urology (2022) recommended the collection of at least 8 systematic biopsy cores in prostates with a size of <30 mL and 10- to 12-core biopsies in larger prostates [43]. Ravery et al. [44] demonstrated that TRUS-guided biopsies using either 10 or 12 cores (if total prostate volume was >50 mL) was associated with 6.6% higher cancer detection compared to the sextant biopsy method, especially in patients with a PSA level under 10 ng/mL and/or a prostate volume over 50 mL. In another study, the addition of four lateral cores at the base and mid-gland as well as two additional mid-lobar, parasagittal transition zone biopsies in case of prostates exceeding 50 mL increased cancer detection rate by 20% compared to sextant biopsy. Moreover, Lee et al. [45] demonstrated the dependency of systematic biopsy sensitivity on the number of biopsy cores taken. In their study, gradual decrease in the mean number of cores from 21.8 to 14.5, 10.9, 7.3, and 5.4 led to a statistically significant reduction in overall csPCa detection rates in non-targeted systematic biopsy by 16%, 13%, 9%, and 8%, respectively, in the entire cohort. In turn, Kwon et al. [42] demonstrated that the 20-core template outperformed the 10-core one in detecting clinically significant cancers in the entire cohort (49% vs. 41%, *p* = 0.02) and in the biopsy-naïve group (48% vs. 40%, *p* = 0.05); however, additional cores did not yield higher detection of Grade Group 1 (GG1) cancers (20-core: 35% vs. 10-core: 44%, *p* = 0.09). Furthermore, more clinically significant cancers were identified in the 12-core trans-perineal biopsy group compared to the 12-core TRUS biopsy (46% vs. 38%, *p* < 0.001). The direction of biopsies is also crucial. PCa is most likely located at the apex and base of the peripheral gland, making these sites the primary targets for biopsies, whereas midline biopsies have the lowest likelihood of positive results [18,46,47]. Various studies have indicated that the addition of laterally directed biopsies, which also sample the lateral horn, increases sensitivity of cancer detection by approximately 5% to 35% [18,46,47]. Most additional cancers were found in the far-lateral mid-lobar region, an area effectively sampled by laterally directed sextant biopsy. A systematic review of 87 studies involving 20,698 patients showed that 12-core prostate biopsy schemes, which add laterally directed cores to the standard sextant scheme, achieved a balance between cancer detection rates and adverse events [15]. Current EAU-EANM-ESTRO-ESUR-ISUP-SIOG-guidelines (2024) present data in favor of collecting a minimum of 12 samples bilaterally from apex to base, as far posterior and laterally as possible, within the peripheral gland, irrespective of the approach used in patients with no previous prostate imaging [34].

### 2.1. Impact of Systematic Biopsy Result on Treatment

As focal therapy gains interest, accurate identification of all tumor foci through comprehensive prostate examination becomes crucial in guaranteeing optimal patient selection and suitable ablation of all regions harboring csPCa [45]. The presence of csPCa foci in untreated regions poses a challenge to the oncological efficacy of focal therapy as a standalone strategy. Post-treatment prostate biopsies have revealed out-of-field recurrences in 4–49% of cases, which may indicate their initial under-detection at MRI imaging and subsequent biopsy, or the development of de novo multifocal disease within the prostate [48,49]. It seems that the former scenario is more probable in patients in whom the post-treatment biopsy was made within 6–12 months of focal therapy initiation. Thus, a comprehensive pre-treatment prostate biopsy may be an underutilized tool in patient selection for focal therapy. Lee et al. [45] also stressed the pivotal role of systematic biopsy (SB) in patient selection for focal therapy. Their results demonstrated that decreasing the number of cores collected during SB significantly increased the percentage of patients necessitating changes in their treatment plans compared to full systematic biopsy. The proportion of patients with altered treatment plans rose from 12% to 29% when comparing a strategy in which the mean number of non-targeted systematic cores was 14.5 to a strategy based on 5.4 cores. The standard 12-core systematic biopsy may be insufficient in case of individuals opting for focal therapy, and therefore saturation biopsy may prove to be a better option for this group. Based on the results of study which employed SB with a median of 23 cores for a median prostate volume of 43.5 mL, Lee et al. [45] suggested a sampling intensity of at least 1 core for every 2 mL of prostate volume as the optimal approach. However, comprehensive SB may not be well-tolerated in patients undergoing transrectal biopsy under local anesthesia, given the related risks of urinary retention and sepsis [45]. Moreover, the detection of significant cancers in non-targeted SB biopsy may be associated with the underestimation of the index lesion [50]. The impact of these smaller foci of untreated disease outside the index lesion on long-term oncological outcomes remains uncertain, and thus require further analysis.

### 2.2. Advantages of Systematic Biopsy in Detecting Prostate Cancer

PCa is multi-focal tumor in up to 80% of men, and systematic biopsy plays a crucial role in identifying foci beyond the index lesion, which may not be adequately visualized on MRI [45,51,52]. SB increases the likelihood of detecting all cancerous areas within the prostate, thereby reducing the risk of misclassifying a higher-risk patient as low-risk [53,54]. By sampling multiple regions of the prostate, it provides a thorough initial assessment of tumor characteristics, including grade, volume, and distribution [55]. This baseline information is critical for accurately stratifying patients for active surveillance programs. The study assessing the distance between cancer-containing systematic samples and MRI-visible lesions revealed that 90% of systematic biopsies comprising csPCa cores were situated within a 10-mm radius of the nearest MRI-visible lesion [56]. A proportion of 18% of csPCas were positioned beyond this 10-mm radius; however, their yield decreased as the distance from the region of interest (ROI) increased. Consequently, the clinical utility of systematic biopsy may lay in capturing perilesional csPCa when ROIs shows negative results. Focusing systematic biopsies around ROI targets, termed targeted regional biopsy, presents a potentially efficient alternative to traditional 12-core templates. It has been demonstrated in one of studies that systematic biopsies performed ipsilaterally to the ROI yielded significantly higher cancer detection rates compared to contralateral biopsies, even in cases where a targeted core showed no cancer [57,58]. Moreover, systematic biopsy appears to be essential to rule out csPCa in magnetic resonance imaging (MRI)-negative regions and confirm the presence of a discrete lesion suitable for focal therapy [59]. However, the value of systematic biopsies covering only areas non-targeted by MRI remains uncertain due to the fact that both biopsies may overlap suspicious regions [60]. Lee et al. [61] demonstrated a csPCa detection rate of 21% for non-targeted systematic biopsy, indicating that excluding systematic biopsy cores overlapping with MRI lesions would only result in a 3% missed detection rate for csPCa. Some studies have indicated that systematic biopsy might decrease the risk of underestimation the Gleason score (GS) [53,54]. Furthermore, collecting cores from areas surrounding a suspicious lesion (focal systematic biopsy) may reduce the risk of sampling error and potentially provide a more accurate estimation of the Gleason score [62]. Moreover, regular systematic biopsies during active surveillance help detect any changes in tumor grade or volume, ensuring timely intervention if the disease progresses [55]. The number of targeted cores taken per lesion affects the detection of csPCa and the estimation of the Gleason score [63]. Specifically, the current standard of obtaining three to four cores from high-volume lesions, such as PIRADS 5, may not be enough to accurately estimate the Gleason score [64]. SB provides detailed information on the location and extent of tumors within the prostate which allows surgeons to tailor the extent of lymph node dissection to individual patient risk profiles. This approach helps in maximizing the therapeutic benefit while minimizing unnecessary surgical intervention [65]. The local staging of prostate cancer is vital for planning nerve-sparing surgery, as it helps surgeons avoid the critical anatomical structures, such as neurovascular bundles, while ensuring complete cancer removal [66]. Accurate identification of the tumor’s proximity to the neurovascular bundles in SB aids in balancing the need for oncological control with the preservation of erectile function and urinary continence [44]. Thus, SB helps in minimizing the risk of the abovementioned postoperative complications by providing a clear map of the prostate and surrounding structures. The extent of PCa involvement in systematic cores may also serve as a significant prognostic indicator of biochemical progression risk or pelvic lymph node involvement. Omitting systematic biopsy cores may lead to erroneous qualification or disqualification for extended pelvic lymph node dissection [67,68].

Available evidence suggests that the omission of systematic biopsy would increase diagnostic uncertainty and worsen risk assessment, since it can translate into 5–16% of csPCa cases that may be overlooked [12,69,70,71]. The results of the GÖTEBORG-2 trial indicated that the omission of systematic biopsies decreased the detection of inPCa by 50%; however, at the same time, one in five csPCa were overlooked [72]. In general, systematic biopsies enabled the recognition of smaller tumors belonging to the intermediate-risk group (ISUP 2) that were not reported while using other methods in this trial. While considering the omission of systematic biopsy, it should be kept in mind that multiparametric magnetic resonance imaging (mpMRI) shows limited sensitivity in identifying all csPCa lesions, with approximately 30% of such lesions being undetectable on mpMRI [73]. mpMRI alone has limitations in predicting local stage and extra-prostatic extension [41,42]. In patients with higher Prostate Imaging Reporting and Data System (PI-RADS) scores, increased possibility of csPCa presence beyond the index lesion has been reported, with lesion outside the index in up to 60% of PI-RADS 5 cases [74,75]. Krausewitz et al. [12] suggested that benefits related to SB in csPCa detection were greater in patients with PI-RADS 3 lesions and increased inversely with PI-RADS grading. In turn, a Polish retrospective study of 225 patients with PCa demonstrated that omission of SB in PI-RADS 5 lesions was associated with a significant decrease in the csPCa detection rate by 6.9% [76]. Moreover, Cochrane meta-analysis of 18 studies revealed that the added value of SB in csPCa detection in biopsy-naive patients was 4.3% [4]. Similarly, in the 4 M trial and MRI-FIRST studies the estimated added value of systematic biopsy was approximately 5% [69,77]. Systematic biopsy can be performed in case of non-suspicious mpMRI results in patients with high prostate-specific antigen density (PSAD) values (>0.15 ng/mL^2^) due to the significantly increased risk of csPCa detection [78]. PSAD is an important predictor that enhances csPCa detection rates in PI-RADS 5 patients. Moreover, PSAD and PI-RADS scores have been found to complement each other in csPCa detection [76]. Malewski et al. [76] reported that omitting systematic biopsy at high PSAD values increased the risk of missing csPCa from 4.7% to 7.4%. In turn, the absolute detection rate of csPCa associated with the use of systematic biopsy increased from 8.1% to 26.5% for the PSAD threshold of 0.17 ng/mL^2^. High PSAD values have been suggested to be associated with larger tumor volumes and a higher likelihood of underestimating the Gleason score [79,80]. Therefore, in cases of high PSAD values, systematic biopsy should not be omitted in PI-RADS 5 lesions.

The available literature indicates that systematic biopsy, when added to targeted biopsy in the initial biopsy, markedly improves csPCa detection compared to repeat biopsy. The risk of missing csPCa when systematic biopsy is omitted appears to be lower in repeat biopsy compared to initial biopsy (3.5% versus 10%, respectively) [76]. Similarly, Cochrane meta-analysis confirmed that added value of systematic biopsy in repeat biopsy is marginal (~2.3%) [81]. The European Association of Urology recommendations also suggest that systematic biopsy may be omitted in repeat biopsy settings, despite weak evidence [82]. The American Urological Association guidelines suggest performance of a systematic biopsy in patients with both an absence of suspicious findings on MRI and an elevated risk for GG2+ prostate cancer (moderate recommendation, evidence level: grade C), as well in those with indications for a repeat biopsy who do not have a suspicious lesion on MRI (conditional recommendation; evidence level: grade B) [36]. In turn, in biopsy-naïve patients with a suspicious lesion on MRI, or in patients with a suspicious lesion on MRI undergoing repeat biopsy, targeted biopsies of the suspicious lesion should be performed and a systematic template biopsy may also be performed (moderate recommendation, evidence level: grade C) [36]. However, SB alone can be considered in patients with a negative PIRADS score (1–2) and low clinical suspicion of PCa (PSA density < 0.20 ng/mL/cc, negative DRE findings, no family history) and also when MRI is not available and a risk calculator indicates the need to perform a biopsy [34].

### 2.3. Limitations and Drawbacks of Systematic Biopsy

Despite its widespread use, systematic biopsy has some limitations, primarily related to inadequate sampling of specific prostate regions, such as the apical, middle, and anterior areas, which often result in false-negative outcomes and consequent underdiagnosis of csPCa [83,84]. Indeed, the results of various studies have demonstrated that the use of this procedure may result in the omission of csPCa as well as the overdiagnosis of inPCa and thus to potential overtreatment-related harms [85,86,87]. This might explain the limited benefits of radical treatment observed in randomized trials, such as PROTECT and PIVOT, both of which utilized systematic TRUS biopsy for cancer diagnosis [88]. According to some studies, the rate of false-negative results associated with initial systematic 12-core biopsy is 20–24%, while others show that this rate may reach up to 49% (saturation biopsy) [89,90,91]. Moreover, up to 40% of low-risk patients with a Gleason score of 3 + 3 during SB showed greater scores on postoperative pathology [92,93].

Numerous passes of biopsy needles through rectal mucosa and the increasing prevalence of drug-resistant bacterial strains translates into higher risk of sepsis and hospitalization rates following transrectal biopsy as well as greater patients’ morbidity [24]. It has been observed that urinary tract infection and prostatitis are more frequent in 18-core biopsies compared to 12-core biopsies [25]. These complications are associated with increased healthcare costs associated with antibiotic prophylaxis to prevent infection, as well as the expenses related to treating post-biopsy infection and sepsis.

Furthermore, transrectal (TR) prostate biopsy has been associated with other severe complications, including rectal bleeding, hematuria, fever, and acute urinary retention [94,95,96]. Rectal bleeding and hematuria, which are typically self-limiting complications, tend to resolve within several days. However, in patients receiving anticoagulant medications, the bleeding may become serious [33]. To reduce the risk of severe bleeding, in such patients anticoagulation therapy should be discontinued at least one week before prostate biopsy. The reduction in the number of biopsy cores could also potentially decrease bleeding complications arising from the procedure, such as bladder tamponade [97]. A high number of biopsy cores have been demonstrated to affect the trajectory of radical prostatectomy and contribute to greater blood loss [98]. Given the high rates of false negatives and complications associated with systematic TR prostate biopsy, the trans-perineal (TP) approach was introduced to enhance biopsy detection rates and safety. A systematic review and meta-analysis including 11 studies and 2569 patients demonstrated that the trans-perineal approach significantly reduced the risk of complications, such as rectal bleeding and fever, while the transrectal approach was associated with less severe patient pain [33]. Despite the administration of enemas before transrectal prostate biopsy, it is still associated with significantly higher risk of infection compared to the trans-perineal approach. Therefore, trans-perineal prostate biopsy is suggested in patients predisposed to infection, such as those with prostatitis, diabetes, or urinary catheterization, to minimize the likelihood of sepsis and severe post-procedural fever [33]. In turn, the disadvantages of trans-perineal approach include low risk of urinary retention requiring catheter placement (in 1.4% of patients), inability to pass urine, hematuria (in 16.0%), hematospermia lasting up to three months (in most men), perianal abscess (in 0.008%) and temporary erectile dysfunction (in less than 5%) [99].

## 3. Emergence and Advancement of Targeted Biopsy Techniques

The development of mpMRI has revolutionized PCa diagnosis by offering superior imaging modalities, including T2-weighted images, diffusion-weighted images (DWIs), and dynamic contrast-enhanced (DCE) images, thus facilitating precise delineation of suspicious lesions [14]. The introduction of PI-RADS version 2 in 2015 enabled the standardization of mpMRI results interpretation and reporting, as well as facilitating risk stratification before biopsy decisions [16]. MRI has emerged as a valuable tool for identifying suspicious areas within the prostate, facilitating targeted biopsies while minimizing unnecessary procedures in men with no detectable lesions [100,101]. The utilization of mpMRI has been found to enhance csPCa identification without the risk of overdiagnosis [71]. This technique accurately defines lesion areas suitable for targeting and, due to the integration of image fusion technology, it simplifies real-time targeted biopsy procedures. Targeted biopsies (TB) offer the advantage of focusing on mpMRI-detected lesions with lower differentiation, thereby mitigating the likelihood of under-grading. Consequently, MRI-targeted biopsies are gaining interest as adjuncts to or substitutes for SB in cases of previously negative biopsies, as initial biopsies, or during active surveillance [4]. A prospective clinical trial which assessed whether using mpMRI before SB would enhance the detection of csPCa in biopsy-naive patients demonstrated that an additional 5% ISUP2 cases could be identified with the addition of SB, but only 1% of these cases were Gleason 4 + 3 or worse [69]. Thus, it appears that, while performing mpMRI before biopsy in biopsy-naive patients shows the potential to improve the detection of csPCa, SB should not be avoided. European Association of Urology guidelines (2020) recommended the mpMRI procedure before every biopsy; however, current guidelines strongly suggest not using MRI as an initial screening tool [34,82]. Moreover, limited biopsy without MRI is suggested in men with suspicion of locally advanced disease on DRE and/or PSA>50 ng/mL, or individuals in whom curative treatment in not an option. However, an MRI procedure should be performed before prostate biopsy in men with suspected organ confined disease (strong recommendation),

### 3.1. Types of MRI-Targeted Biopsy

MRI-targeted biopsy (MRI-TB) methods, which include cognitive biopsy, MRI in-bore guided biopsy and MRI-ultrasound (US) software fusion biopsy, can be executed via transrectal or trans-perineal approaches [87]. Such biopsies are selectively conducted upon the identification of suspected lesions indicative of clinically significant prostate cancer on MRI scans [4].

#### 3.1.1. Perilesional Biopsy (Figure 1)

Perilesional biopsy involves taking core samples from the area surrounding a lesion at various distances from the lesion. This method can help identify cancer cells around the lesion [102]. The diagnostic yield for csPCa could be improved by including perilesional sampling within a 10-mm radius from the ROI, potentially making “targeted regional biopsy” a standard approach [56]. Theoretical support for the importance of perilesional biopsy comes from the work of Priester et al. [103], which showed that MRI lesions typically underestimate actual cancer diameter by 11 mm and volume by three-fold. This finding was later confirmed by other studies. Brisbane et al. [56] demonstrated that 25% of csPCa cores lie within a 10-mm radius outside the MRI-identified lesion. The combination of systematic and perilesional biopsy was found to be associated with the risk of missing 7% of significant cancers, and for PIRADS ≥ 4 lesions the risk was 5% [102]. Another study showed that that the TB plus perilesional biopsy within a 10 mm radius detected 92% of csPCa cases [104]. Tschirdewahn et al. [105] found that this method identified 99% of csPCa cases. Combination of targeted and systematic cores within a 10 mm radius from the MRI lesions detected 90–92% of csPCa, while utilizing a 15 mm radius enabled the detection of 94–97% [56,104]. The distance required to encompass 90% of csPCa may vary with the PI-RADS score of the lesion: 5.5 mm for PI-RADS 5, 12 mm for PI-RADS 4, and 16 mm for PI-RADS 3 [56]. Moreover, Diamand et al. [106] revealed that perilesional sampling reduces the upgrading events in final pathology compared to systematic or targeted biopsies alone. The PI-RADS v2 Steering Committee recommends performing the biopsy of both the ROI and the surrounding perilesional tissue for PI-RADS 4 and 5 lesions [107]. According to current European Association of Urology, perilesional sampling should complement targeted biopsy in patients with MRI positive (i.e., PI-RADS ≥ 4) results (weak recommendation) [34]. Targeted biopsy with perilesional sampling can also be considered when MRI is indeterminate (PI-RADS = 3), and clinical suspicion of PCa is very low (PSA density < 0.10 ng/mL/cc, negative DRE findings, no family history).

**Figure 1:**
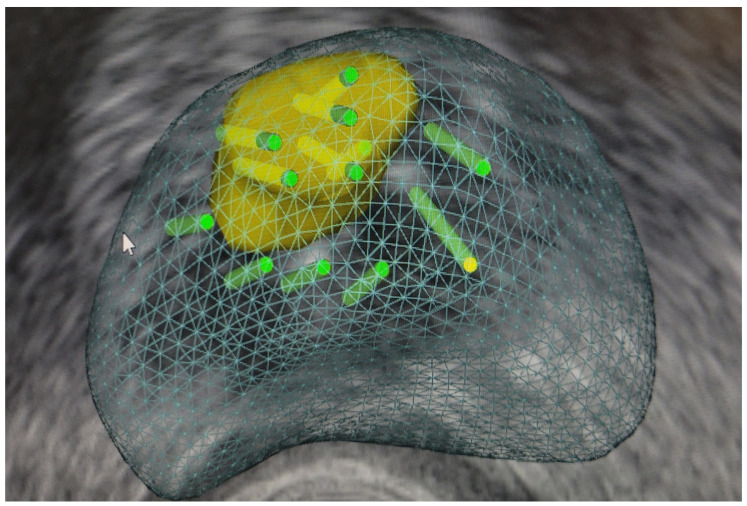
Targeted fusion prostate biopsy with perilesional cores.

#### 3.1.2. Cognitive Biopsy

Cognitive biopsy is based on utilizing MRI findings to identify suspicious regions within the prostate, followed by targeted biopsy under US guidance but without employing software registration of MRI and US images. This technique, also referred to as visually directed biopsy or cognitive fusion biopsy, relies on the operator’s interpretation of MRI to identify suspicious lesions, then using anatomical landmarks to guide biopsy to the site corresponding to US images. While feasible via transrectal or trans-perineal routes, the latter approach offers advantages, such as consistent prostate positioning for both MRI and biopsy. Moreover, this technique enables concurrent systematic biopsies. Nonetheless, cognitive biopsy’s reliance on operator proficiency poses challenges, particularly for individuals lacking experience in prostate MRI or US-guided biopsy. Difficulty in cognitive registration may arise in the absence of identifiable landmarks or in cases of large prostate glands with small targets, as well as in the presence of substantial calcifications obscuring the target [87].

#### 3.1.3. Fusion Targeted Biopsy (Fus-TB) (Figure 2)

The combination of MRI with ultrasound fusion targeted biopsy (fus-TB) shows the strengths of both MRI and ultrasound technologies, as it enables real-time targeted biopsies guided by fusion software, thereby enhancing diagnostic accuracy [14]. This procedure is suitable for clinical settings since it does not need dedicated hardware [108]. The software aligns the lesion identified on MRI with a corresponding location on US to guide the biopsy needle to the targeted site [87]. This technique can be adapted to both transrectal and trans-perineal approaches. Registration between MRI and US can be accomplished using either rigid method, which corrects for rotational discrepancies or the elastic registration method, compensating for deformations of the prostate caused by adjacent structures or the US probe [109,110].

**Figure 2:**
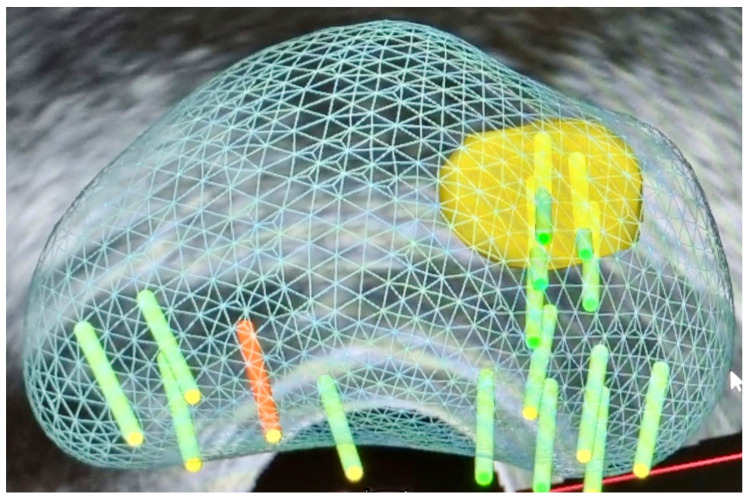
Targeted biopsy with systematic cores.

Benefits of fus-TB include the possibility of documenting biopsy sites for patient follow-up during active surveillance and for quality assurance purposes [87]. Moreover, it shows high efficiency in accurately determining patients’ true pathological grades, thereby decreasing the risk of potential misclassification of inPCa and contributing to reduction in both overtreatment and undertreatment by minimizing diagnostic uncertainties [14,111,112,113]. Moreover, fus-TB addresses challenges encountered in targeting lesions not visualized by US or obscured by significant calcifications. It also holds potential as a substitute for the traditional 12-core SB, particularly in cases with prostate PI-RADS scores of 4 and 5 identified by mpMRI [114,115].

However, the drawbacks include upfront system acquisition costs, longer duration of the biopsy compared to cognitive biopsy, and the risk of technical problems during the procedure, such as failure to upload segmented images for targeting or successful MRI-US image registration. Additionally, the multi-step nature of the technique, involving MRI and US segmentation, image registration, and target sampling, is susceptible to operator-dependent errors, which can compromise the accuracy of target registration between modalities [87].

#### 3.1.4. MRI In-Bore Biopsy

The in-bore biopsy technique is available with 1.5-T or 3-T MRI systems and can be conducted under moderate sedation or with the administration of rectal lidocaine gel. In-bore biopsy ensures precise needle placement at the target site through a transrectal approach (most commonly) but also via a trans-perineal or trans-gluteal approach [116]. In the transrectal approach, a rectal sleeve needle with a trocar is inserted, and the system software guides adjustments in needle direction and length based on MR images. After confirming correct needle placement, imaging is repeated to detect any sampling issues due to patient movement or anatomical changes. In turn, in the trans-perineal approach, a needle-guide template is fixed against the perineum. Then, the system software recognizes the target and the correct hole in the template for needle insertion, followed by adjustments until proper positioning is confirmed. The use of a robotic template for trans-perineal MRI in-bore biopsy has been reported to enhance needle placement accuracy compared to manual templates, but at the expense of significantly prolonged procedure durations (90–100 min) and considerable costs associated with robotic implementation [117]. Compared to systematic or other targeted biopsy methods, in-bore biopsy requires fewer overall cores [116,118]. However, this procedure cannot be conducted in an office setting and is more time-consuming [119,120].

### 3.2. Transrectal Versus Trans-Perineal Fusion Biopsy

MRI-targeted biopsy based on cognitive, fusion, or MRI in-bore techniques, can be conducted through transrectal or trans-perineal approaches. The transrectal approach positions the patient typically in the left lateral decubitus position (for cognitive and fusion techniques), or in prone position (for MRI in-bore procedures) [119]. The transrectal approach typically utilizes local anesthetic. Although the periprostatic nerve block greatly enhances peri-operative pain management when compared to the use of an anesthetic gel applied intrarectally, this method was suggested to potentially introduce or spread pathogens due to additional needle insertions through the rectum [121,122]. However, an updated meta-analysis of 28 RCTs with 4027 patients found no evidence that use of periprostatic injection for local anesthesia resulted in more infectious complications than no injection (RR: 95% CI: 1.08 [0.79–1.48]) [123].

In the transrectal approach, a needle guide is fixed to the probe, which is then inserted into the rectum to obtain biopsy specimens from the prostate through the rectal wall. The needle goes through either the transverse or sagittal plane of the prostate, primarily collecting the sample within its posterior part. However, the transrectal approach may disturb registration between MR and ultrasound images due to the oblique angulation of the transrectal probe [87]. Moreover, the risk of sepsis is the primary concern with the transrectal approach, with infection rates ranging from 0.7% to 7.0% [124,125,126]. To avoid such complication, antibiotics (e.g., fluoroquinolones) are administered; however, their common use can contribute to bacterial resistance [127]. Moreover, the use of fluoroquinolones has been found to be associated with various adverse effects, including tendon rupture, soft-tissue injuries and tendinitis, as well as aortic dissection and rupture [128,129]. Due to the increase in fluoroquinolone resistance, the European Commission has enforced strict regulations on the use of fluoroquinolones, leading to the suspension of their use for peri-operative antibiotic prophylaxis, including for prostate biopsy [130]. Current evidence indicates that in countries permitting the use of fluoroquinolones for antibiotic prophylaxis, it is recommended to administer a full day’s course or to use targeted therapy if fluoroquinolone resistance or augmented prophylaxis (combination of two or more different classes of antibiotics) for patients undergoing transrectal biopsy [131]. In patients in whom trans-perineal access is not possible, rectal swab followed by stool culture should be performed pre-biopsy. Such an approach enables the use of individual targeted antimicrobial therapy [131]. In countries where fluoroquinolones are not allowed for prostate biopsy prophylaxis, alternatives, such as cephalosporins, aminoglycosides, or Fosfomycin, can be used [131]. Moreover, various rectal preparation practices for transrectal biopsies, including enemas, povidone-iodine chlorhexidine applications and bowel preparation, are used [123]. The results of systematic review and meta-analysis demonstrated that povidone-iodine for rectal preparation before a prostate biopsy significantly decreased the risk of infection (RR 0.50, 95% CI 0.38–0.65) and the likelihood of hospitalization (RR 0.38, 95% CI 0.21–0.69). Another study showed the reduction in infection rate following the insertion of an iodophor cotton ball before placing an ultrasonic probe to the rectum during TRUS-guided prostate biopsy [132]. As a result, the trans-perineal approach, associated with a lower infection rate of 0.6%, is more and more frequently utilized [133,134]. However, as aforementioned, a recent randomized study demonstrated that transrectal biopsy with targeted prophylaxis, requiring rectal cultures and meticulous attention to the selection and administration of antibiotics, resulted in similar infection rates [35]. Targeted antibiotic prophylaxis, which relies on rectal swab and urine culture, is essential for preventing severe infections after rectal prostate biopsy [135]. However, a limitation of this approach is that screening for bacteria with resistance to a single antibiotic, such as cotrimoxazole, is not cost-effective due to the wide variety of bacterial colonies in the rectum. Therefore, routine screening generally targets multi-resistant strains, which does not eliminate the possibility of detecting bacteria resistant to only one antibiotic [136].

The trans-perineal approach carries a lower risk of hematochezia but a potentially higher risk of urinary retention depending on core sampling density [137]. Cognitive, fusion, and MRI in-bore biopsy using trans-perineal biopsies are usually conducted with the patient in the lithotomy position and the biopsy needle traverses the prostate in the craniocaudal plane from apex to base. Despite the trans-perineal approach, a transrectal ultrasound probe is still necessary for prostate imaging in cognitive and fusion biopsies. Brachytherapy grip and a stepper to stabilize it against the perineum may also be employed [138].

### 3.3. Number of Cores Per Target

The quantity of targeted cores obtained per lesion during targeted biopsy is important as it may affect the detection rate of csPCa and the estimation of Gleason scores [63]. Collection of only three to four cores (which is currently a standard practice) from high-volume lesions (e.g., PIRADS 5) may prove insufficient for precise estimation of Gleason scores [64]. Furthermore, the mpMRI technique has its limitations, including limited ability to predict extra-prostatic extension and local staging [76,139,140]. Consequently, relying solely on TB results may affect clinical decision-making processes, including the choice of local treatment or extension of surgical intervention.

Apart from the mpMRI, diverse ultrasound imaging techniques, such as contrast-enhanced ultrasound, computer-assisted TRUS, histoscanning, and sonoelastography are under development along with evaluation for effectiveness in localizing suspicious prostate lesions. Nevertheless, these methods require further refinement before potential integration into routine clinical practice [141].

## 4. Comparative Analysis of Systematic Biopsy vs. Other Diagnostic Techniques (Table 1)

The determination of the optimal biopsy strategy for patients presenting with suspected PCa lesions on MRI remains a subject of ongoing debate. SB has been suggested to be associated with a higher likelihood of overlooking aggressive PCa variants compared to fus-TB or combined biopsy (comPB) approaches, thereby posing a risk of undertreatment [142]. MpMRI of the prostate, including T2-weighted imaging, dynamic contrast enhancement, diffusion-weighted imaging (DWI) and interpretation using PI-RADS or a Likert scale, has demonstrated superior detection of csPCa and reduced identification of inPCa compared to standard transrectal biopsy [16,81,101,143,144]. Studies including men referred for biopsy due to clinical suspicion of prostate cancer have revealed that TB guided by MRI findings lead to lower rates of detecting inPCa compared to standard biopsies, with similar or enhanced detection rates for csPCa [55,69,145]. A systematic review and meta-analysis of 68 studies with a paired design and eight randomized clinical trials (RCTs) and a total of 14,709 men who received MRI-TB or SB or both demonstrated that MRI-TB more efficiently identified csPCa compared to SB (detection ratio [DR] 1.16 [95% confidence interval (CI) 1.09–1.24], *p* < 0.0001) as well as less inPCa (DR 0.66 [95% CI 0.57–0.76], *p* < 0.0001). The proportion of cores positive for cancer was also higher for MRI-TB than for systematic biopsy (relative risk (RR) 3.17 [95% CI 2.82–3.56], *p* < 0.0001). Moreover, adding SB to MRI-TB increased the likelihood of detecting insignificant cancer by 34% [145]. The GÖTEBORG-2 trial also demonstrated that utilization of TB alone decreased the detection of insignificant carcinomas [72].

**Table 1:** Detection rates of clinically significant prostate cancers by systematic and targeted prostate biopsy.

Study	Systematic Biopsy	Targeted Biopsy
Abd Ali, F., et al. [5]	75%	77%
Rouvière, O., et al. [69]	14%	20%
van der Leest, M., et al. [77]	25%	23%
Drost, F.-J.H., et al. [81]	21,16%	18.9%
Ahmed, H.U., et al. [101]	51%	49%
Klotz, L., et al. [144]	30%	35%
Kasivisvanathan, V., et al. [137]	43%	41%
Kasivisvanathan, V., et al. [112]	26%	38%
Porpiglia, F., et al. [146]	44%	18%
Tonttila, P.P., et al. [147]	64%	67%
Valerio, M., et al. [148]	24%	33%

The outcomes from randomized controlled trials among biopsy-naïve subjects provide conflicting evidence regarding the superiority of MRI-TB over SB in detecting csPCa [112,146,147]. The results of the PRECISION study revealed that MRI followed by MRI-TB enabled the identification of more csPCa with fewer diagnoses of inPCa in men exhibiting an MRI-visible lesions [112]. Similar reduction of inPCa detection was demonstrated in the MRI-FIRST study [69]. Some studies revealed that MRI-targeted biopsy substantially enhanced cancer detection rates in individuals with prior negative biopsies but not in biopsy-naïve individuals [92,149].

In turn, Pezelj et al. [150] demonstrated that patients who received a TB exhibited a diminished incidence of under-grading. Although the proportion of positive cores between the mpMRI and non-mpMRI was comparable (35.3% versus 34.0%, *p* = 0.09), the TB group demonstrated a higher number of positive cores (4.6 vs. 3.4, *p* = 0.03). A greater amount of sampled material potentially facilitates more accurate tumor grading by pathologists [150]. Moreover, the prospective observational study demonstrated comparable detection rates in TB and SB; however, TB appeared to be more sensitive to intermediate- and high-grade tumors [5]. TB exhibited superior discriminatory efficacy in identifying PCa with ISUP 2 or higher, demonstrating a sensitivity of 90% and an 80% correct assignment rate. However, TB failed to detect nearly one-third of ISUP 1 tumors, among which 20% were csPCa based on current guidelines [5]. This limitation seems acceptable due to the typically indolent nature of ISUP 1 tumors, as evidenced by autopsy data indicating that a substantial proportion of men have undiagnosed PCa without compromising life expectancy. Moreover, recent research suggests delaying treatment for selected patients with intermediate-risk PCa, although definitive criteria for patient selection remain elusive [148,151]. It has been suggested that MRI-targeted biopsy may overlook some “MRI-invisible” low-volume ISUP 2 disease; however, the studies demonstrated that this type of cancer exhibits distinct biological characteristics compared to MRI-visible csPCa [152]. Overall survival for men with non-visible Gleason 3 + 4 reflected that of men with Gleason 3 + 3 disease, while men with MRI-visible prostate Gleason 3 + 4 cancer showed much worse outcomes [153]. Consequently, it has been suggested that the clinical relevance of Gleason 3 + 4 cancer identified via SB may differ from that detected via MRI-TB [85]. In turn, Nakanishi et al. [154] demonstrated that TB missed only 4.6% of csPCa cases in patients with PI-RADS 5 scores; however, it also missed as many as 22% of cases in men with PI-RADS 3 and 4 scores. Bryk et al. [57] observed a notable enhancement in the detection of csPCa with the addition of six ipsilateral SB to targeted biopsies. Conversely, contralateral SB primarily detected insignificant disease [57]. The problem of potentially overlooking the most representative regions within the target, including the so-called “penumbra”, is of high importance [107]. Recently, van der Leest et al. [77] demonstrated that incorporating four perilesional cores (“focal saturation”) improved csPCa detection. These findings indicate the potential for reduction in SB if both the lesion and surrounding tissue are adequately sampled, as suggested by PI-RADSv2.1 guidelines, which advocate for targeting both the lesion and its perilesional ‘penumbra’ [64]. Notably, all ‘target saturation biopsy methods’ are effective in diminishing potential targeting errors by surgeons or fusion software and in addressing the underestimation of lesion size on MRI [64].

In turn, the comparison of software-based three-dimensional-guided systematic prostate biopsy (3D-GSB) and conventional TRUS to assess PCa detection rates in 956 patients without prior positive biopsies and with a PSA value ≤ 20 ng/mL demonstrated no significant differences in the clinically csCDR between the 3D-GSB and TGSB groups (33.3% vs. 28.8%, *p* = 0.385) [155]. However, in this study, the overall CDR was significantly higher for 3D-GSB compared to TRUS (55.6% vs. 39.9%, *p* = 0.002). In addition, 3D-GSB detected significantly more non-significant PCa cases than TRUS (22.2% vs. 11.1%, *p* = 0.004). Moreover, in patients with PCa, TRUS yielded a significantly higher number of cancer-positive SB cores compared to 3D-GSB (42% vs. 25%, *p* < 0.001). Thus, in the opinion of the authors, at present, 3D-GSB does not seem to provide added value to conventional TRUS.

Additionally, fus-TB presents a favourable profile by increasing csPCa detection rates while reducing biopsy core requirements. While some studies report comparable overall PCa detection rates between fus-TB and SB, others highlight the superiority of the first in detecting csPCa [156,157]. One study demonstrated the superior capability of a combination of mpMRI with targeted fusion biopsy in detecting csPCa, compared to transrectal SB [71]. Vourganti et al. [158] also demonstrated that fus-TB enabled the identification of PCa in 34–37% of patients with previously negative SB, a third of whom exhibited a Gleason score of ≥7. In turn, the results of a meta-analysis and systematic review revealed no significant discrepancies in overall PCa detection rates between fus-TB and SB among patients with at least one prior negative biopsy [14]. However, subgroup analyses demonstrated a lower PCa detection rate with fus-TB in biopsy-naive patients compared to SB. Furthermore, subsequent analyses revealed that fus-TB significantly outperformed SB in detecting PCa in patients undergoing trans-perineal fus-TB; however, fus-TB did not exhibit superiority over SB in patients undergoing transrectal fus-TB [14]. It appears that the trans-perineal approach enhances overall PCa detection rates by targeting commonly missed anatomical sites, such as the apical, dorsolateral, and anterior prostatic segments [159]. A meta-analysis demonstrated that the trans-perineal approach to software fusion biopsy yielded a higher detection rate of both csPCa and anterior tumors, as well as a lower complication rate compared to the transrectal approach [160]. The trans-perineal approach also offers lower infection risks compared to transrectal methods. The superiority of fus-TB in detecting csPCa may be dependent upon sample size, as fus-TB did not outperform SB in detecting csPCa in subgroups with ≤100 samples. Moreover, the sequence of biopsy procedures may influence detection rates, with studies suggesting that performing SB first may reduce subsequent fus-TB accuracy due to tissue alterations induced by bleeding and oedema [161,162]. Conversely, performing fus-TB first may diminish the csPCa detection rate of SB as a result of the removal of tissue within clinically significant area. Moreover, the results of a systematic review and meta-analysis based on eight studies and 2603 men with suspected PCa demonstrated the non-inferiority of PCa diagnosis by the combination of TB + regional biopsy approach, thus suggesting the potential for the replacement of SB with FSB [64].

Many studies show the higher sensitivity of TB in identifying csPCa compared to standard biopsy however, due to the fact that they analysed only populations referred for biopsy based on clinical suspicion, the translation of findings to population-based screening appears challenging since, in such population, the majority of individuals referred to biopsy is expected to have lower risk of csPCa [163]. Table 1 presents selected trials and a meta-analysis with detection rates of significant prostate cancer by systematic and targeted biopsy.

## 5. Integration Strategies: Harmonizing Systematic and Targeted Biopsy

The MRI-based approach using the Prostate Imaging Reporting and Data System (PI-RADS) in prostate cancer evaluation has been demonstrated to enhance detection of csPCa, decrease inPCa identification, and lower the number of biopsies compared to systematic biopsy [77,101]. However, the use of MRI alone results in the omittance of a substantial amount of csPCa [4]. To ensure proper sampling of an MRI-detected lesion, a minimum of three to five cores is necessary [164,165]. Using additional perilesional or regional systematic biopsies, instead of standard sextant-based systematic biopsies, can reduce the total number of cores by avoiding systematic biopsies in MRI-negative lobes, as well as ameliorate the detection of csPCa by compensating for guidance imprecision [34]. Combined biopsy approaches, including MRI-TB and SB or fus-TB and SB, have also emerged as promising strategies to improve diagnostic efficacy, particularly in detecting csPCa [166,167]. Deng et al. [14] observed that comPB significantly augmented both overall PCa and csPCa detection rates compared to fus-TB alone. One of the meta-analyses emphasized the superiority of comPB over individual biopsy modalities in detecting csPCa [168]. Similarly, Krausewitz et al. [12] demonstrated a considerable rise in the detection rates of PCA and csPCa through combined biopsy techniques. The cumulative CDR was as high as 72%, closely resembling the results of the PRECISION trial’s CDR (71%) [112]. The authors observed that all biopsy methods when performed alone showed the underestimation of true tumor grading [12]. Other studies also indicated higher consistency with pathological tumor grading for combined biopsy compared to SB and MRI-TB [12,55]. Moreover, Krausewitz et al. [12] suggested that omitting SB could decrease unnecessary core sampling in nearly two-thirds of cases and reduce the number of men diagnosed with inPCa. In contrast, TB alone was associated with significantly improved efficiency in PCA and csPCa detection compared to SB and combined biopsy in overall and PI-RADS-dependent analyses (*p* < 0.001 for all), suggesting the potential to reduce biopsy-related anxiety, distress, pain, and risk of infections through a TB-limited approach [169]. However, such an approach was associated with a misdiagnosis rate of 7.4% for csPCa and 4.7% for cancers graded ≥ ISUP 4. Similarly, a comprehensive systematic literature search and analysis revealed that TB alone in MRI-positive men missed the diagnosis in 17.2% of men with ISUP grade 2 or higher PCA [4]. In turn, a combined approach including TB and focal perilesional saturation biopsy (FSB) has been suggested to decrease misdiagnosis rates of TB alone for csPCa and high-risk PCA by 92.3% and 80%, respectively [12]. However, the use of SB instead of FSB may result in a missed and/or underestimated csPCa rate of 4.7% and high-risk PCA rate of 8.5%. A proportion of 92% of misdiagnosed csPCa cases overlooked by FSB are located on the contralateral prostate lobe [64,105].

The combination of MRI-TB and SB during initial investigation is suggested by some authors; however, the precise contribution of SB to TB remains debatable [4,101,170,171]. Some experts believe that a combined approach can improve true tumor grading and that of extent, thus reducing the likelihood of misdiagnosis [55,69,172]. However, lack of certainty regarding tumor grading persists after the combined approach in one-third of cases [55]. It has been also implied that the specific reason underlying the enhanced performance of the additional combined approach could possibly be associated with the compensation for targeting errors, under-sampling of the target approach and MRI-invisible cancers, considering the multifocality of prostate cancer lesions [171,173]. The combination of lesion-targeted and SB leads to a greater yield of csPCa compared to either of these methods alone [17,55,56,71,166].

## 6. Conclusions

Precise risk stratification plays a pivotal role in the management of PCa patients, as it helps to lower the risk of overtreatment of inPCa as well as undertreatment of csPCa, thereby improving patients’ quality of life and optimizing treatment outcomes [14]. The results of recent studies demonstrate a shift towards a more selective approach in detecting high-risk cancers that need therapy, while adopting conservative methods in case of less aggressive tumors [5]. Guided biopsies are increasingly recommended due to their potential to enhance the detection of high-risk prostate cancer while reducing the identification of low-risk cases [174]. A Lower under-grading rate due to diminished sampling error further favors the rationale for adopting this technique as the standard in prostate cancer detection [14]. The trend towards MRI-directed management, typically involving mpMRI followed by MRI-targeted biopsy (MRI-TB), has resulted in increased utilization of MRI-TBs, prompting the standardization of MRI-TB techniques [87,175]. Isolated MRI-TB emerges as a suitable strategy for initial biopsy in biopsy-naïve individuals and those undergoing active surveillance (AS) [5]. Omitting additional SB decreases the incidence of septic complications and local inflammation, potentially improving subsequent surgical outcomes, but may also decrease the cancer detection rate. Therefore, the choice of biopsy strategy should be tailored to the individual patient’s risk profile, with a combined approach of targeted biopsies and at least perilesional sampling offering optimal sensitivity for detecting csPCa. Future advancements in imaging technologies and biopsy techniques, including artificial intelligence-augmented lesion detection and robotic-assisted sampling, hold promise for further enhancing the specificity and efficacy of PCa detection [176].

## List of Abbreviations

PCa	Prostate cancer
csPCA	clinically significant prostate carcinoma
inPCA	insignificant prostate cancer
MRI	magnetic resonance imaging
ISUP	International Society of Urological Pathology
PSA	Prostate-specific antigen
DRE	digital rectal examination
TRUS	transrectal ultrasound
CDR	cancer detection rates
GG	Grade group
SB	systematic biopsy
ROI	region of interest
GS	Gleason score
mpMRI	multiparametric magnetic resonance imaging
PI-RADS	Prostate Imaging Reporting and Data System
PSAD	prostate-specific antigen density
TR	transrectal
TP	trans-perineal
DWI	diffusion-weighted images
DCE	dynamic contrast-enhanced
TB	targeted biopsy
fus-TB	fusion targeted biopsy
comPB	combined prostate biopsy
RCT	randomized clinical trials
DR	detection ratio
CI	confidence interval
RR	relative risk
3D GSB	three-dimensional-guided systematic prostate biopsy (3D-GSB)
FSB	focal perilesional saturation biopsy
AS	active surveillance
